# Cadaveric analysis of surgical techniques and working space for retroperitoneal tumors as model for improving resection of neuroblastoma

**DOI:** 10.1186/s12893-024-02508-x

**Published:** 2024-07-31

**Authors:** Grigore Cernaianu, Greta Franke, Nora Elena Kühne, Miriam Meurer, Ralf-Bodo Trobs, Frank Eifinger, Martin Dübbers, Martin Scaal, Reza Vahdad

**Affiliations:** 1 Division of Pediatric Surgery, University Hospital Cologne, Kerpenerstr. 62, 50937 Köln, Germany; 2 Department of Pediatric Surgery and Pediatric Orthopedics, St. Hedwig Clinic, Steinmetzstraße 1-3, 93049 Regensburg, Germany; 3 Department of Pediatric Critical Care Medicine and Neonatology, University Hospital Cologne, Kerpenerstr. 62, 50937 Cologne, Germany; 4 Department of Anatomy, Faculty of Medicine, University of Cologne, Joseph-Stelzmann Str. 9, 50931 Köln, Germany; 5 Department of Pediatric Surgery, University Hospital of Marburg, 35043 Baldingerstraße, Marburg, Germany

**Keywords:** Surgery, Technique, Cadaveric, Neuroblastoma, IDRF

## Abstract

**Purpose:**

Neuroblastoma, the most common extracranial solid tumor in children under 5 years, often surrounds visceral arteries. This study aimed to analyze the working space provided by standardized surgical techniques at key arterial landmarks in adult cadavers.

**Methods:**

We assessed in eight adult cadavers the mobilization of the left colon, spleen and pancreas, right colon, duodenum and mesenteric root, access to the bursa omentalis. The average working space score (AWSS) was evaluated at the left and right renal artery, left and right side of the coeliac trunk, superior mesenteric and common hepatic artery. The score was defined as: (0) vessel not visible, (1) working space at the vessel ≤ 1x diameter of the aorta, (2) < 3x the diameter of the aorta, (3) ≥ 3x diameter of the aorta.

**Results:**

The maximum AWSS of 3 was achieved at key vascular landmarks through specific mobilization techniques.

**Conclusion:**

Additional mobilization of spleen, pancreas and mesenteric root and access to the bursa omentalis increase surgical working space at major visceral arteries. The results of our investigation provide surgeons with a useful guide to prepare for abdominal neuroblastoma resection.

## Introduction

Retroperitoneal tumors with encasement of aorta, coeliac trunk, superior mesenteric artery and renal arteries pose a surgical challenge. This challenge has prompted research to optimize the exposure tactics in both adults and children [1–8]. Neuroblastoma is an embryonal sympathetic nervous system tumor and the most frequent extracranial solid tumor in children, affecting 10.2–10.9 cases per million children in the USA and Europe [9]. 95% of all neuroblastomas occur in children below 5 years of age [10]. 48% of primary tumors arise in the adrenal gland and 25% in the extra adrenal retroperitoneum [11]. 40% of patients present with metastatic disease and, despite myeloablative high-dose chemotherapy, surgery and radiotherapy, only up to 64.6% survive 5 years after diagnosis [12].

Gross-total resection of > 95% of the tumor has been shown to be associated with increased survival in patients with large and metastatic tumors in numerous recent publications [13–15].

**Fig. 1:**
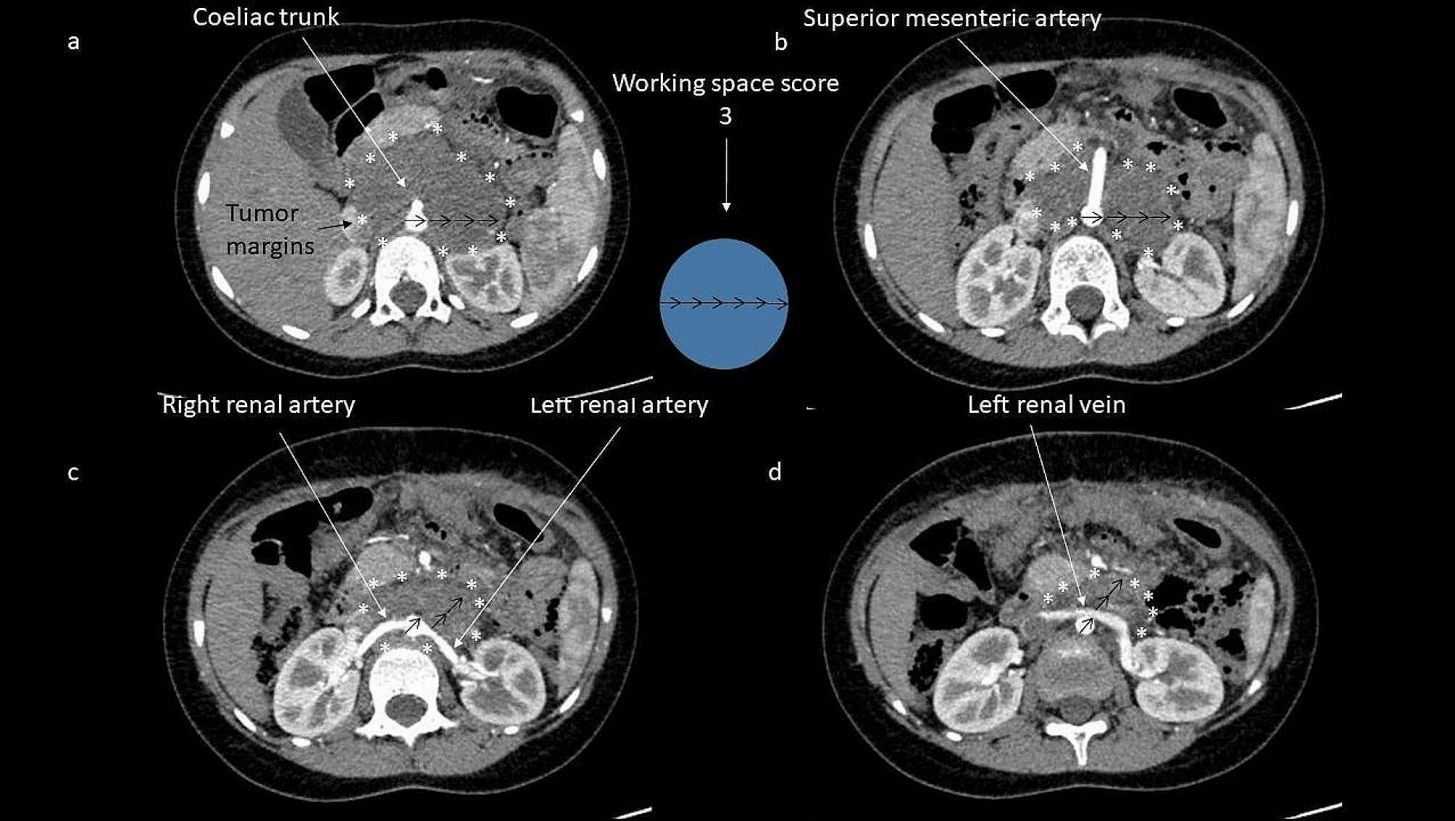
Preoperative computed tomography after application of intravenous contrast substance of a child following induction chemotherapy with persistent vascular encasement of major abdominal arteries by neuroblastoma Borders of the tumor (starlets) and expected working space as a multiple of the aortal diameter (arrows) required to see the tumor margin at the level of the (**a**) coeliac trunk, (**b**) superior mesenteric artery, (**c**) right renal artery, (**d**) left renal vein. The circle depicts the desired circular maximum working space from the center of the landmark vessel equaling a radius of 3x the diameter of the aorta (working space score 3)

The surgical complexity of this tumor lies in the anatomic challenges posed by involvement of major arteries (Fig. 1) [16, 17]. Two classification systems are commonly used to decide whether patients undergo upfront surgery or neoadjuvant induction chemotherapy.

The older International Neuroblastoma Staging System (INSS) [18] discerns tumors crossing the midline of the abdomen from those not crossing the midline (defined as tumors beyond the opposite border of the vertebral column). It is commonly used, when assessing the cross-sectional radiologic anatomy before surgery. However, this classification neither takes into account, whether the tumor originates from the right or left side nor the surgical techniques best suited to expose the invaded area.

The newer International Neuroblastoma Risk Group Staging System (INRGSS) [11, 12] takes the extent of adherence of the tumor to vessels into account. It bases on image-defined risk factors (IDRF) [11, 12, 16, 19–21], such as encasement of the superior mesenteric artery, of the coeliac trunk and its branches, or contact to the renal vessels.

The presence of IDRFs is associated with more intraoperative complications, reduced likelihood of complete resection and, ultimately, decreased overall survival [22–26]. However, the INRGSS does not reflect the size or the origin of the tumor.

Increasingly, even children without metastases but presenting IDRFs, are subjected to induction chemotherapy, in an attempt to improve resectability [25, 27]. However, despite some shrinkage of the tumor, even after 2–3 months of delay to surgery, the IDRFs remained unchanged in 49% of patients [19].

In consequence, the proper choice of surgical exposure techniques is of paramount importance to maximize the view around the affected vessels, in order to achieve maximal resection and safety [9].

A number of surgical exposure techniques for the retroperitoneal regions have been well-described in pediatric surgery [6, 8], as well as vascular, visceral, transplant and urologic adult surgery [2, 3, 5, 28–31]. The key techniques are the medial visceral rotations [3–5]. These are standardized surgical techniques intended to rotate the abdominal organs to the midline of the abdomen in order to expose the retroperitoneal anatomy. The right medial visceral rotation consists of mobilization of the right-sided colon, duodenum and mesenteric root (technique according to Cattell-Braasch) [4]. The left medial visceral rotation consists of two separated elements, mobilization of the left colon and mobilization of spleen, pancreas and stomach (technique according to Mattox) [3, 32]. For the abdominal anatomic region located above the renal vessels and between aorta and inferior vena cava, techniques to access the omental bursa are used as a standard during resection of pancreatic tumors in adult visceral surgery [30, 31].

However, to the authors´ knowledge, up to date, there is no anatomic study which analyzes the working space in a specific anatomic region, depending on the used surgical exposure techniques. In consequence, for abdominal neuroblastoma, there is no evidence-based surgical algorithm adapting surgical exposure to tumor invasion.

The aim of this cadaveric study was to analyze which combinations of established surgical exposure techniques achieve an optimal working space around major visceral arteries representing IDRFs in abdominal neuroblastoma.

## Methods

Vascular exposures were performed on eight adult human cadavers from the Department of Anatomy (four females). The age of the cadavers ranged from 67 to 100 years. During their lifetime, the donors had willed their cadavers to the Department of Anatomy and consented to participate post mortem in medical research and education. The experimental cadaveric study design was approved by the institutional ethics review board of the University Hospital of Cologne (Approval number 18 − 012). Seven cadavers were conserved with formaldehyde and one cadaver was fresh frozen. The evaluation of the working space obtained with standardized surgical exposure techniques were performed by two specialist pediatric surgeons and a medical student.

### Step 1 - anatomic regions

**Fig. 2:**
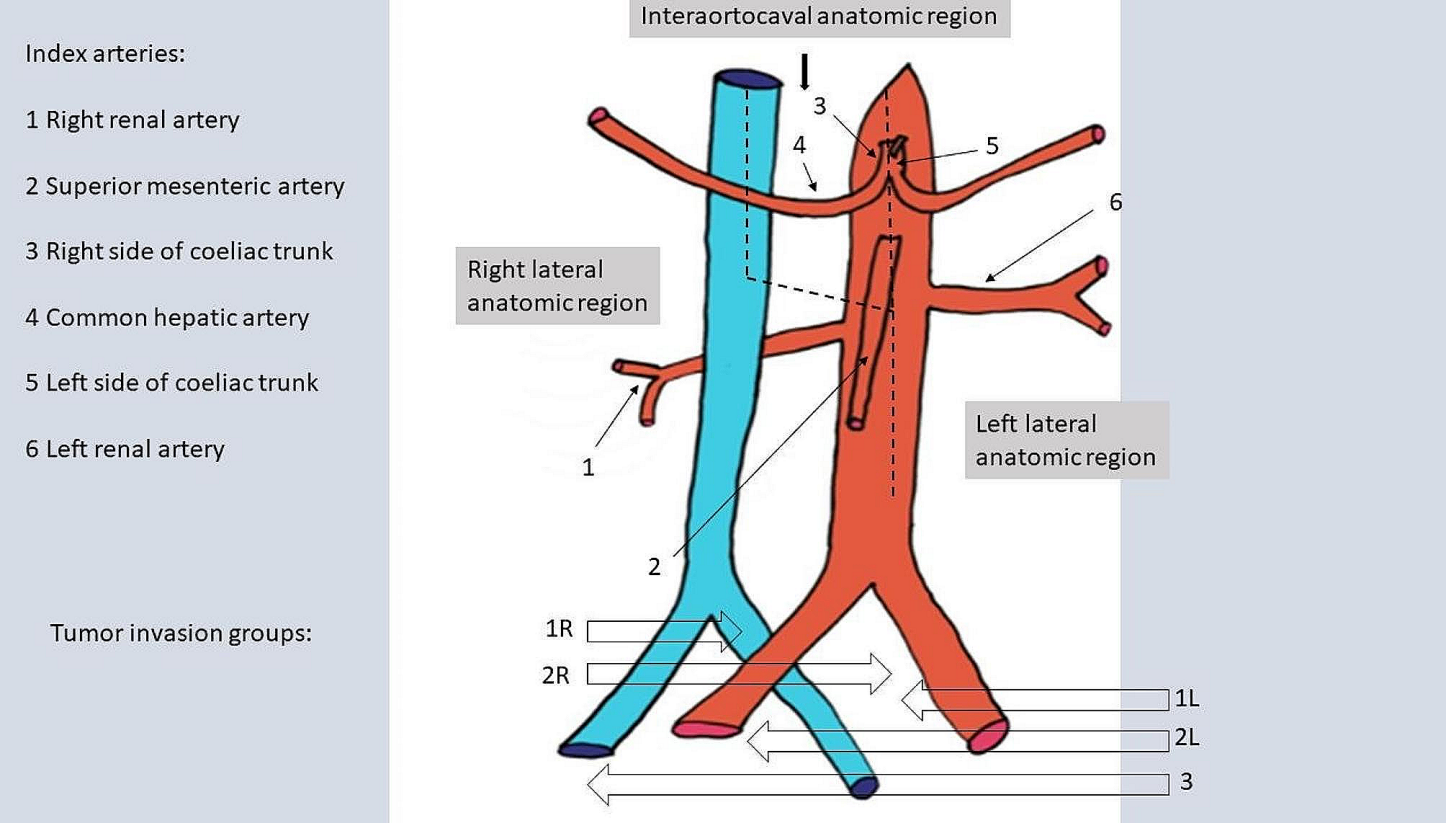
Anatomic regions, vascular landmarks and tumor invasion groups Margins of the anatomic regions (dashed lines). Index arteries: left lateral anatomic region: at the left side of the coeliac trunk and the left renal artery; interaortocaval anatomic region: at the common hepatic artery and the right side of the coeliac trunk; right lateral anatomic region: at the right renal artery and the superior mesenteric artery. Tumor invasion groups: tumor isolated in right lateral anatomic region (**1R**), tumor isolated in left lateral anatomic region (**1L**), tumor invading right lateral + interaortocaval anatomic region (**2R**), invading left lateral + interaortocaval anatomic region (**2L**), tumor invading right lateral + interaortocaval + left lateral anatomic region or isolated in the interaortocaval anatomic region (3)

We divided the abdomen into three anatomic regions (Fig. 2). The right lateral anatomic region is located above and until the level of the origin of the superior mesenteric artery between the right flank and the ventral circumference of the inferior vena cava. It follows then downward an S-shaped course to the origin of the superior mesenteric artery and continues further downward along the ventral circumference of the aorta. This definition reflects the extended access available on the right side of the retroperitoneum by mobilization of the ascending colon and duodenum. The left lateral anatomic region is limited between the left flank and the ventral circumference of the abdominal aorta. The interaortocaval anatomic region is located above and until the level of the origin of the superior mesenteric artery between the ventral circumferences of the inferior vena cava and aorta.

### Step 2 - image-defined vascular landmarks within the anatomic regions

Specific anatomic segments of six major visceral arteries served as index arteries for each anatomic region. These segments are commonly identified at cross-sectional imaging. For the right lateral anatomic region (1) the right renal artery and (2) the origin and the distal portion of the superior mesenteric artery served as index arteries. For the interaortocaval region, we used (3) the right part of the coeliac trunk and (4) the common hepatic artery as vascular landmarks. For the left lateral region, the landmarks were (5) the left part of the coeliac trunk and (6) the left renal artery.

### Step 3 - surgical exposure techniques

The laparotomy and the division of the falciform ligament and the right and left hepatic coronary ligaments for maximal mobilization of the liver started the surgical procedures. In contrast to our usual clinical approach consisting of a transverse laparotomy, we had to perform an additional median laparotomy in order to overcome the rigidity of the cadavers.

Subsequently, we performed four surgical exposure techniques in a specific order. Following every surgical procedure technique, the working space obtained at each of the six vascular landmarks was scored. This resulted in 24 scores for every cadaver.

The first surgical exposure step was the mobilization of the descending colon. Subsequently, the additional mobilization of the spleen and pancreas was performed as the second surgical procedure and another set of scores was established. The mobilization of spleen and pancreas was achieved by division of the spleno-renal ligament, subsequently developing a plane dorsal of the splenic vessels. By this step, the spleen and tail as well as the body of the pancreas were medially rotated, thus completing the Mattox maneuver of left-sided medial visceral rotation. After mobilization of the left colon, spleen and pancreas, we left the loops around the origins of the coeliac trunk and superior mesenteric artery in place and then restored the normal position of the left colon, spleen and pancreas before proceeding to the next surgical technique.

The third surgical technique was the mobilization of the right colon, of the duodenum and of the mesenteric root from the ileocolic junction to the ligament of Treitz. Following the diagonal mobilization of the mesenteric root and the subsequent mobilization of the lower border of the horizontal part of the duodenum, the small bowel was lifted up, exposing the mesenteric root. By this approach, the right-sided medial visceral rotation was completed, according to Cattell-Braasch. Each of these elements were investigated as one surgical technique and scored as described above.

Finally, the fourth scored surgical exposure technique was the access to the omental bursa through an upper exposure via longitudinal incision of the lesser omentum and a lower exposure via additional incision of the gastro-colic ligament under the greater omentum.

### Step 4 - working space score

After performing each of the four surgical exposure techniques in a cadaver, the members of the team preliminarily assessed the working space at each of the six vascular landmarks. The working space score was defined as a circle with the center at the vascular landmark. The radius of the working space was related to the diameter of the aorta as reference. Photographs were taken and the diameter of the aorta was marked with a line using PowerPoint^®^ software (Microsoft, Seattle, USA). The line was digitally copied, thus ensuring the preservation of its size, and added on the photograph from the target vessel in the center to the margin of the visible working space field. The working space score was (0) if the vessel segment was not visible with this exposure technique, (1) if the working space at the vessel segment was ≤ 1x diameter of the aorta. We attributed a score of (2) if the working space at the vessel segment was > 1 but < 3x the diameter of the aorta. Finally, we attributed a score of (3) if the working space at the vessel segment was ≥ 3x diameter of the aorta.

Following completion of all surgical techniques in a cadaver, the view obtained with every technique was reassessed until an agreement was reached on the final working space score.

After completing the dissection of all eight cadavers, the average working space score (AWSS) in all eight cadavers and the minimum – maximum score was computed for each of the four surgical techniques at each of the 6 vascular landmarks, resulting in 24 AWSS.

### Step 5 - tumor invasion groups

We then classified tumor invasion groups (Fig. 2), which took into account the areas invaded by the tumor, as visible on radiologic imaging. They discerned between tumors invading only the lateral anatomic regions (group 1), tumors additionally invading the interaortocaval anatomic region but without involving the contralateral lateral anatomic region (group 2) and tumors invading the whole abdomen from the right to the left lateral anatomic region (group 3).

Furthermore, we discerned between tumors with origins on the right side, the left side or on the interaortocaval region of the abdomen.

As a result, we defined tumor invasion group 1R as being limited to the right lateral abdominal compartment and not invading beyond the ventral circumference of the inferior vena cava, tumor invasion group 1 L as being limited to the left lateral abdominal compartment and not invading beyond the ventral circumference of the abdominal aorta, group 2R invading into the interaortocaval region and originating from the right lateral region, group 2 L invading into the interaortocaval region and originating from the left lateral region. Tumor invasion group 3 was defined as extending bilaterally from the left to the right lateral anatomic region or originating in the interaortocaval anatomic region.

### Step 6 - surgical exposure algorithm

Our goal was to create a surgical exposure algorithm, which matches the tumor invasion groups to the best suited surgical exposure techniques. It should translate preoperative radiologic anatomy into intraoperative surgical tactic.

Our approach was to select for every anatomic region the surgical techniques with the highest AWSS with respect to its specific vascular landmarks. The algorithm was subsequently built by adding the techniques with the highest AWSS of all anatomic regions involved in the respective tumor invasion group.

## Results

### Analysis of the average working space score (AWSS) for surgical exposure techniques

#### Mobilization of the left colon

The mobilization of only the left descending colon (Table 1) resulted in each of the eight cadavers in a working space of ≤ 2x diameter of the aorta at the left renal artery. This resulted in an average working space score (AWSS) of 2 at the left renal artery. Furthermore, following the mobilization of only the left colon, the working space at the left origin of the coeliac trunk was < 3x diameter of the aorta in 3/8 cases, resulting in a score of 2. In the other 5/8 cases, we considered the working space at the left origin of the coeliac trunk to be ≤ 1x diameter of the aorta, resulting in a score of 1. In consequence, the AWSS for the left-sided origin of the coeliac trunk was 1.37. The working space in the interaortocaval anatomic region was very reduced. In each of the eight cadavers, the working space at the right side of the coeliac trunk, as well as at the common hepatic artery, was ≤ 1x diameter of the aorta (AWSS 1). This exposure was very limited and not well suited for the exposure of any invasive tumor. The view on the right anatomic region was not suitable for resection of large tumors, as the working space at the superior mesenteric artery was in 5/8 patients ≤ 1x diameter of the aorta and < 3x diameter in 3/8 patients, resulting in an AWSS of 1.37. In addition, we were not able to safely expose the right renal artery, following only mobilization of the left colon (AWSS 0).

**Table 1:** Mobilization of left colon - AWSS

Mobilization of left colon – Working space score						
Anatomical region	Right lateral		Interaortocaval		Left lateral	
Cadaver	Right renal artery	Superior mesenteric artery	Common hepatic artery	Right side of coeliac trunk	Left side of coeliac trunk	Left renal artery
1	0	1	1	1	2	2
2	0	2	1	1	1	2
3	0	1	1	1	1	2
4	0	1	1	1	1	2
5	0	2	1	1	2	2
6	0	1	1	1	1	2
7	0	2	1	1	1	2
8	0	1	1	1	2	2
AWSS (min.-max.)	0 (0–0)	1.37 (1–2)	1 (1–1)	1 (1–1)	1.37 (1–2)	2 (2–2)

Working space score: (0) vessel segment not visible with this exposure technique, (1) working space at the vessel segment ≤ 1x diameter of the aorta, (2) working space at the vessel segment < 3x the diameter of the aorta, (3) working space at the vessel segment ≥ 3x diameter of the aorta. AWSS average working space score in eight cadavers

#### Additional mobilization of spleen/pancreas

**Fig. 3:**
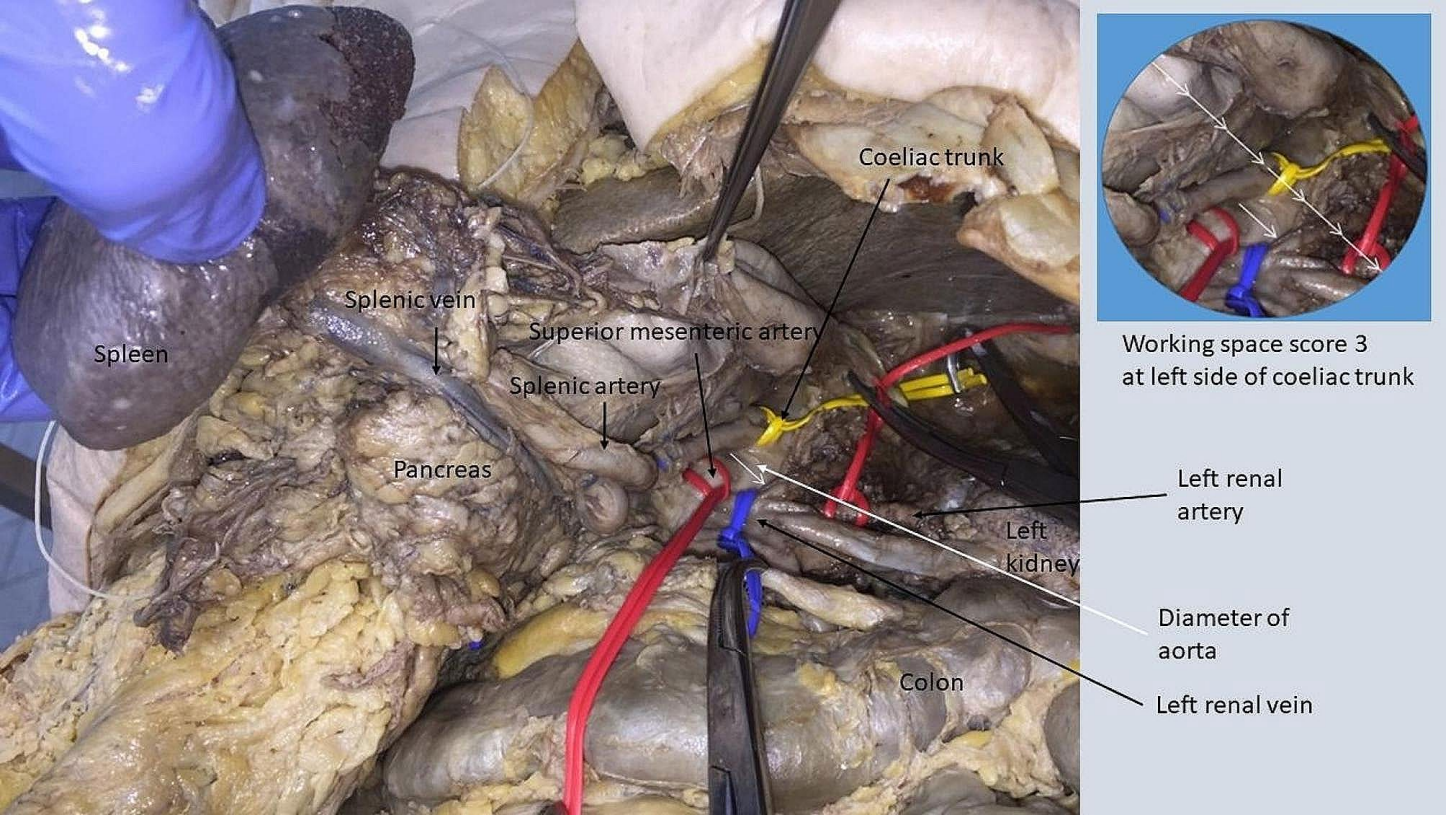
Mobilization of spleen/pancreas The area inside the circle represents the working space with 3x diameter of aorta, equaling a score of 3 at the left side of coeliac trunk

Following additional mobilization of spleen and pancreas (Fig. 3) the working space within the left anatomic region increased (Table 2). The working space was > 3x diameter of the aorta and suitable for resection of large tumors in this region, as it offered a very good working space at the left renal artery (AWSS 3) and at the left side of the coeliac trunk (AWSS 3). We encountered a reduced working space at the right side of the coeliac trunk (AWSS 1), as well as at the common hepatic artery (AWSS 1). The superior mesenteric artery could be exposed with this technique but the working space from the left side of the aorta was scored 2 in 3/8 and 1 in 5/8 cadavers (AWSS 1.37). The right renal artery could not be sufficiently exposed (AWSS 0) following this technique.

**Table 2:** Mobilization of spleen/pancreas - AWSS

Working Space Score After Mobilization of Spleen and Pancreas Following Left Colon Mobilization.						
Anatomical region	Right lateral		Interaortocaval		Left lateral	
Cadaver	Right renal artery	Superior mesenteric artery	Common hepatic artery	Right side of coeliac trunk	Left side of coeliac trunk	Left renal artery
1	0	1	1	1	3	3
2	0	2	1	1	3	3
3	0	1	1	1	3	3
4	0	1	1	1	3	3
5	0	2	1	1	3	3
6	0	1	1	1	3	3
7	0	2	1	1	3	3
8	0	1	1	1	3	3
AWSS (min.-max.)	0 (0–0)	1.37 (1–2)	1 (1–1)	1 (1–1)	3 (3–3)	3 (3–3)

Working space score: (0) vessel segment not visible with this exposure technique, (1) working space at the vessel segment ≤ 1x diameter of the aorta, (2) working space at the vessel segment < 3x the diameter of the aorta, (3) working space at the vessel segment ≥ 3x diameter of the aorta. AWSS average working space score in eight cadavers

#### Mobilization of right colon + duodenum + mesenteric root

**Fig. 4:**
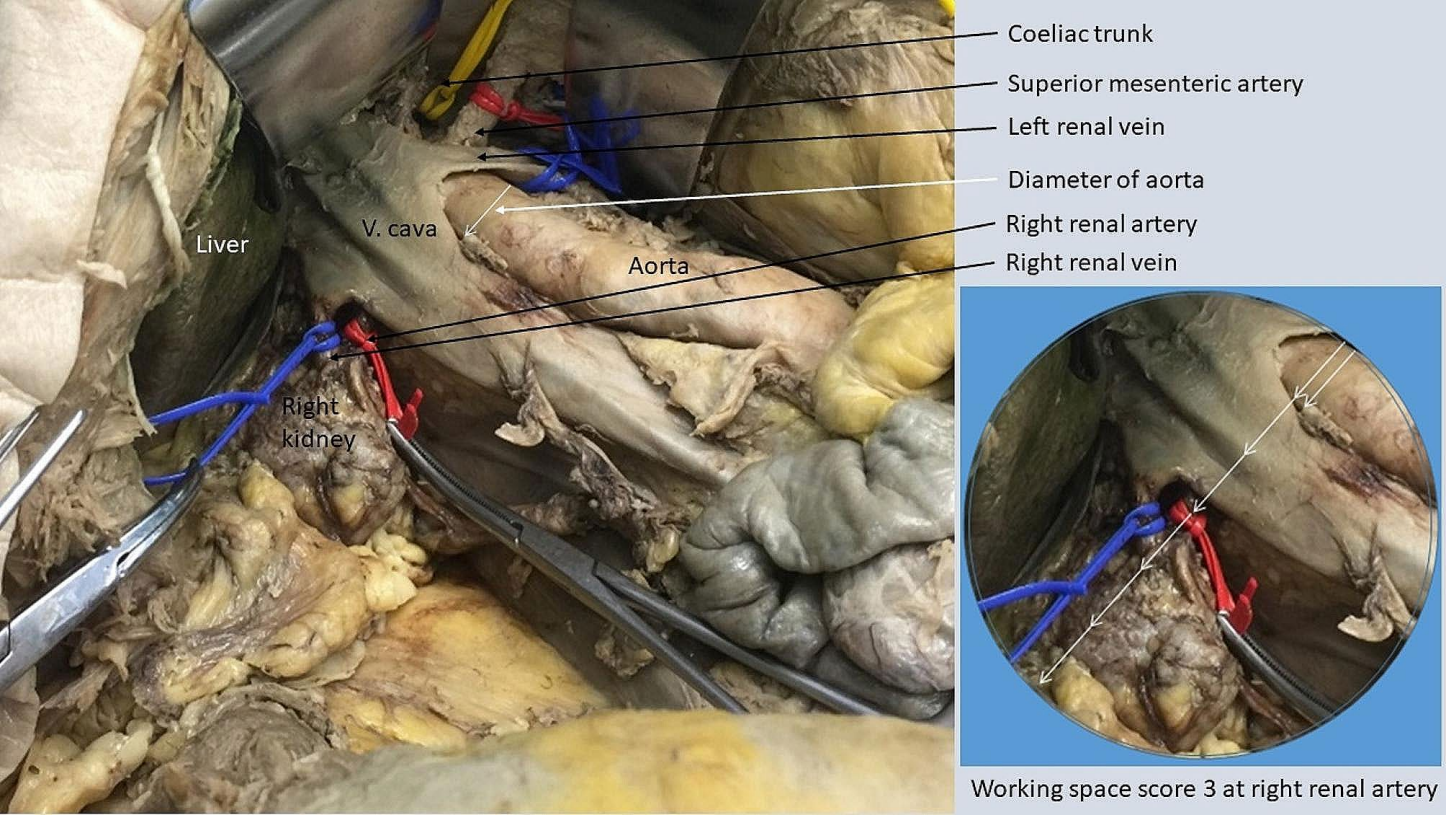
Mobilization of the right colon, duodenum and mesenteric root The area inside the circle represents the size of the working space with 3x diameter of aorta, equaling a score of 3 at the right renal artery

Following mobilization of the right colon, duodenum and mesenteric root (Fig. 4), the working space was very limited at the left renal artery (AWSS 1), the left side of the coeliac trunk (AWSS 1) and the right side of the coeliac trunk (AWSS 1) (Table 3). Even with a vessel loop left in place from the previous mobilization of the left colon, spleen and pancreas, it was difficult to view the coeliac trunk only by the right-sided exposure in the cadaver, even in absence of a tumor. The common hepatic artery could be followed after incision of the hepato-duodenal ligament in its distal part but was barely visible (AWSS 1) and the central part could not be visualized with this technique only. In contrast, the working space was very good at the superior mesenteric artery (AWSS 3), with the vessel being exposed while following the left renal vein as a landmark. This technique offered an excellent working space (AWSS 3) at the right renal artery.

**Table 3:** Mobilization of right colon, duodenum and mesenteric root – AWSS

Mobilization of right colon, duodenum and mesenteric root – Working space score						
Anatomical region	Right lateral		Interaortocaval		Left lateral	
Cadaver	Right renal artery	Superior mesenteric artery	Common hepatic artery	Right side of coeliac trunk	Left side of coeliac trunk	Left renal artery
1	3	3	1	1	1	1
2	3	3	1	1	1	1
3	3	3	1	1	1	1
4	3	3	1	1	1	1
5	3	3	1	1	1	1
6	3	3	1	1	1	1
7	3	3	1	1	1	1
8	3	3	1	1	1	1
AWSS (min.-max.)	3 (3–3)	3 (3–3)	1 (1–1)	1 (1–1)	1 (1–1)	1 (1–1)

Working space score: (0) vessel segment not visible with this exposure technique, (1) working space at the vessel segment ≤ 1x diameter of the aorta, (2) working space at the vessel segment < 3x the diameter of the aorta, (3) working space at the vessel segment ≥ 3x diameter of the aorta. AWSS average working space score in eight cadavers

#### Access to omental bursa

**Fig. 5:**
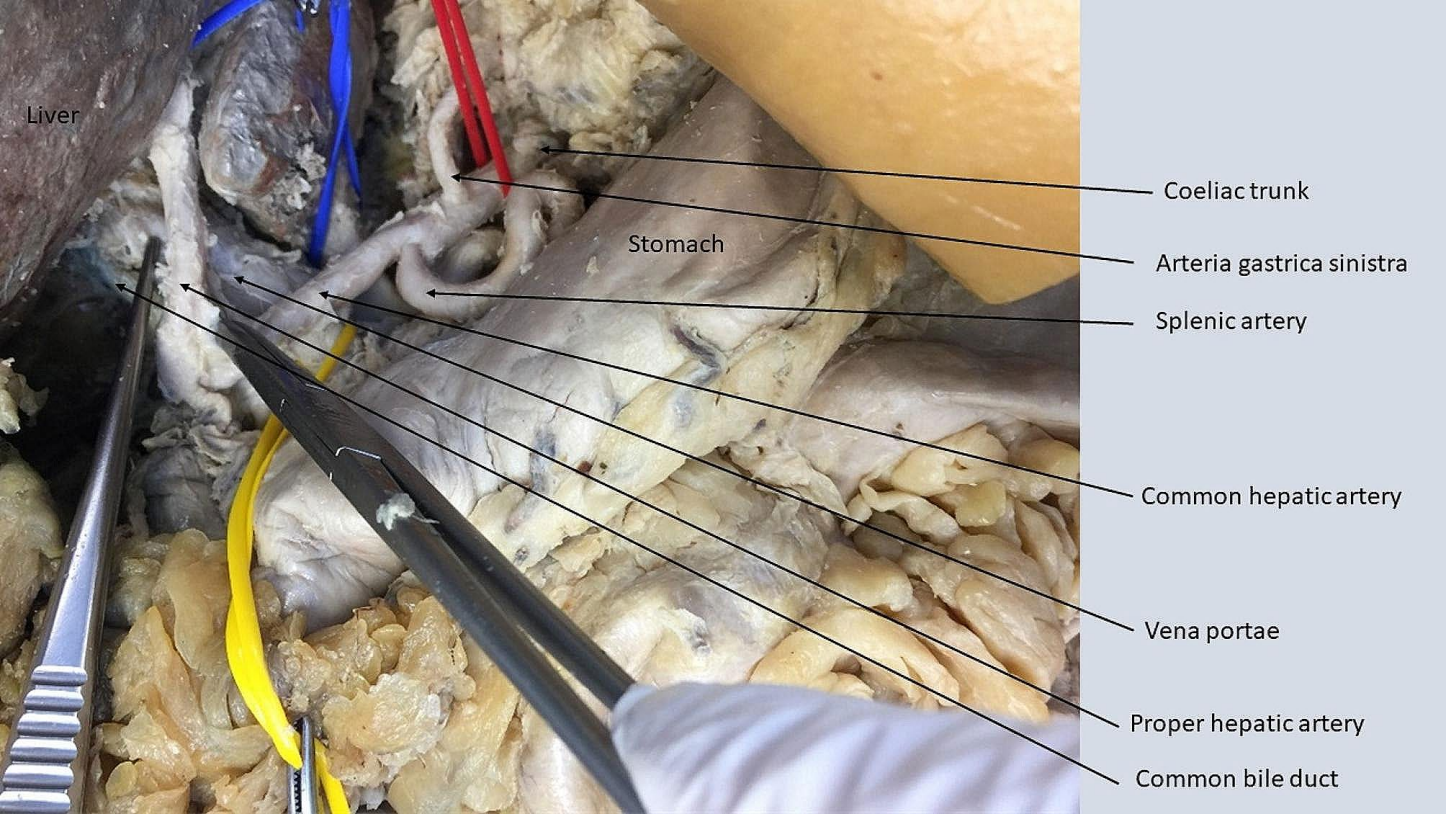
Upper access to bursa omentalis via lesser omentum

**Fig. 6:**
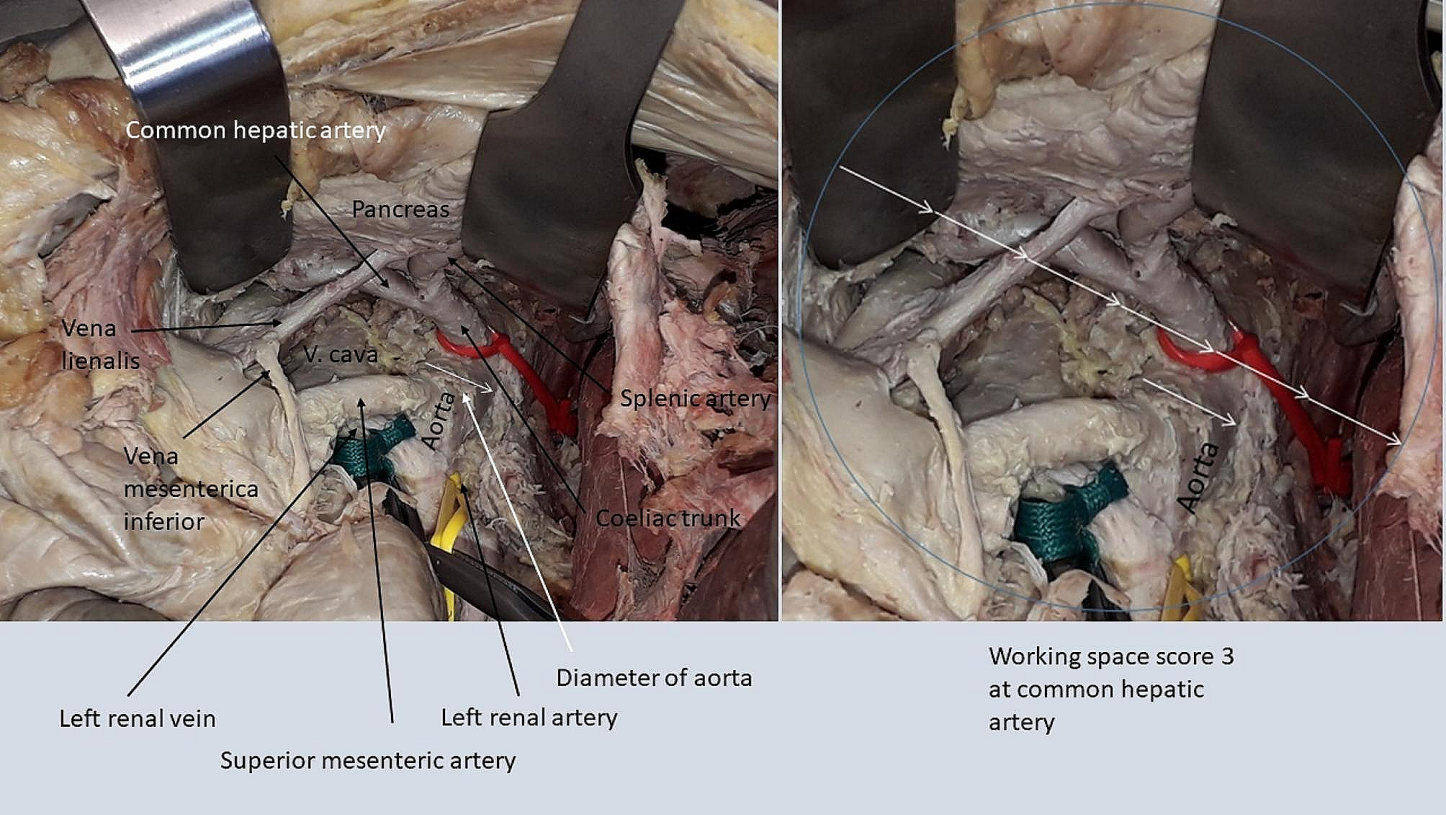
Lower access to the bursa omentalis via the gastro-colic ligament The area inside the circle represents the size of the working space with 3x diameter of aorta, equaling a score of 3 at the common hepatic artery

When accessing the omental bursa, we found an increased working space at the same vascular landmarks being obtained after concomitantly entering the lesser omentum (Fig. 5) and the gastro-colic ligament (Fig. 6).

This technique did not expose the left renal artery (AWSS 0). The left side of the coeliac trunk was scored as 1 in 5/8 patients and 2 in 3/8 patients, resulting in an AWSS of 1.37. Our impression was that most of the working space at the coeliac trunk had been obtained with the previous techniques and that it would have been difficult to resect a tumor at the coeliac trunk following only this technique.

The situation was different in the interaortocaval zone. While the access through the lesser omentum was suitable to expose the central part of the common hepatic artery, the additional access through the gastro-colic ligament increased the working space at this vessel. When access through the lesser omentum and through the gastro-colic ligament were scored as one technique (Table 4), we found an excellent working space at the common hepatic artery (AWSS 3) and at the right side of the coeliac trunk (AWSS 3).

The working space at the superior mesenteric artery was scored 2 in 3/8 cadavers, while being scored 1 in the remaining 5/8 cadavers, thus resulting in an AWSS of 1.37. The right renal artery was not visible (AWSS 0) with this technique.

**Table 4:** Access to omental bursa – AWSS

Access to omental bursa – Working space score						
Anatomical region	Right lateral		Interaortocaval		Left lateral	
Cadaver	Right renal artery	Superior mesenteric artery	Common hepatic artery	Right side of coeliac trunk	Left side of coeliac trunk	Left renal artery
1	0	1	3	3	1	0
2	0	2	3	3	2	0
3	0	1	3	3	1	0
4	0	1	3	3	1	0
5	0	2	3	3	2	0
6	0	1	3	3	1	0
7	0	2	3	3	2	0
8	0	1	3	3	1	0
AWSS (min.-max.)	0 (0–0)	1.37 (1–2)	3 (3–3)	3 (3–3)	1.37 (1–2)	0 (0–0)

Working space score: (0) vessel segment not visible with this exposure technique, (1) working space at the vessel segment ≤ 1x diameter of the aorta, (2) working space at the vessel segment < 3x the diameter of the aorta, (3) working space at the vessel segment ≥ 3x diameter of the aorta. AWSS average working space score in eight cadavers

### Surgical algorithm for maximum working space depending on extent of tumor invasion

Each anatomic region had a surgical exposure technique, which yielded the maximum working space at its vascular landmarks (Table 5). For the left anatomic region, this was the case for the mobilization of the left colon and the additional mobilization of spleen and pancreas. For the interaortocaval region, the access to the bursa omentalis offered the best overview. Concerning the right lateral anatomic region, the best overview was obtained with the mobilization of the right colon, duodenum and mesenteric root.

**Table 5:** Optimal average working space scores (AWSS 3) in the anatomic regions

Average (min.-max.) working space score (AWSS)						
	Anatomic region					
	Right lateral		Interaortocaval		Left lateral	
Surgical technique	Right renal artery	Superior mesenteric artery	Common hepatic artery	Right side of coeliac trunk	Left side of coeliac trunk	Left renal artery
Mobilization of left colon	0 (0–0)	1.37 (1–2)	1 (1–1)	1 (1–1)	1.37 (1–2)	2 (2–2)
Mobilization of left colon, spleen and pancreas	0 (0–0)	1.37 (1–2)	1 (1–1)	1 (1–1)	3 (3–3)	3 (3–3)
Mobilization of right colon, duodenum and mesenteric root	3 (3–3)	3 (3–3)	1 (1–1)	1 (1–1)	1 (1–1)	1 (1–1)
Access to bursa omentalis	0 (0–0)	1.37 (1–2)	3 (3–3)	3 (3–3)	1.37 (1–2)	0 (0–0)

AWSS average working space score in eight cadavers. Working space score: (0) vessel segment not visible with this exposure technique, (1) working space at the vessel segment ≤ 1x diameter of the aorta, (2) working space at the vessel segment < 3x the diameter of the aorta, (3) working space at the vessel segment ≥ 3x diameter of the aorta

In consequence, the following algorithm (Table 6) is suited for maximum surgical working space, based on the tumor invasion group:

Tumors limited to the right lateral region (tumor invasion group 1R) are best exposed by mobilization of right colon, duodenum and mesenteric root (Cattell-Braasch). Tumors limited to the left lateral region (tumor invasion group 1 L) are best exposed by mobilization of left colon, spleen and pancreas.

In case of additional interaortocaval invasion (groups 2R and 2 L), the access to the bursa omentalis is added. Finally, for tumors extending from the left to the right lateral anatomic region (group 3), the optimal exposure is achieved by combining mobilization of right colon, duodenum and mesenteric root (Cattell-Braasch) with access to the bursa omentalis and mobilization of the left colon, spleen and pancreas.

**Table 6:** Surgical exposure algorithm for abdominal tumors

Tumor invasion group	Surgical exposure algorithm for abdominal tumors
1R	Mobilization of right colon + duodenum + mesenteric root (Cattell-Braasch)
1 L	Mobilization of left colon, spleen, pancreas and stomach (Mattox)
2R	Cattell-Braasch and access to bursa omentalis
2 L	Mobilization of left colon, spleen, pancreas and access to bursa omentalis
3	Cattell-Braasch and mobilization of left colon, spleen, pancreas and access to bursa omentalis

Combinations of exposure techniques resulting in an average working space score ≥ 3x diameter of the aorta at all the index arteries within the tumor invasion group. Tumor invasion groups: tumor isolated in right lateral anatomic region (1R), tumor isolated in left lateral anatomic region (1 L), tumor invading right lateral + interaortocaval anatomic region (2R), invading left lateral + interaortocaval anatomic region (2 L), tumor invading right lateral + interaortocaval + left lateral anatomic region or isolated in the interaortocaval anatomic region (3)

## Discussion

The cadaveric setting offered a unique opportunity to assess different techniques exposing major visceral vessels, which are rarely encountered in the operating room apart from major tumor resections. It enabled us to simulate the exposure of the coeliac trunk and its branches, as well as of the superior mesenteric and the renal arteries without fearing complications.

Cadaveric studies are a valuable tool not only to improve surgical skills [33–38] but also to optimize surgical approaches around important anatomic structures in general [39–41], vascular [42, 43], urologic [44–46] and gynecologic [47] surgery. A number of cadaveric studies have been used to assess the anatomic relations between splanchnic nerves and lumbar vessels, which are important for retroperitoneal lymphadenectomy, as in the case of testicular cancer. We adopted the idea to normalize the measurements of the working space around the target vessels to the diameter of the aorta [44, 48]. By expressing the working space as a multiple of the respective aortal diameter as marked on from photographs, we attempted to objectify our results and exclude shrinking artefacts and inter-individual variability exemplified by the different body sizes.

Mobilization of the left descending colon alone did not provide sufficient working space at the left coeliac trunk and left renal artery. Mobilization of spleen and pancreas increased the working space at the left side of the coeliac trunk and the left renal artery. The maximum working space at the right renal artery and the superior mesenteric artery was achieved using the mobilization of the right colon, duodenum and mesenteric root. However, these techniques were not sufficient to achieve a good exposure of the right side of the coeliac trunk and the common hepatic artery. An improved exposure of this region was achieved by adding techniques accessing the omental bursa.

The use of the score allowed us to measure and describe that exposing multiple major visceral arteries can be best achieved using combinations of multiple exposure techniques.

These results are of clinical relevance for neuroblastoma surgery, as image-defined-risk factors frequently involve several of the investigated vessels [19, 22, 24, 49].

The surgical techniques are well described by surgeons of different specialties [2, 3, 6–8, 29], but our work is, to our knowledge, the first study by pediatric surgeons, specifically analyzing the working space required for resection of neuroblastoma in a cadaveric setting. We provide experimental evidence that tumors encasing the coeliac trunk are best exposed with combinations of several surgical techniques instead of mobilization of the colon only on the site where the tumor originated. Apart from the differences between adults and children in anatomical investigations, we feel that our work is applicable to both adult surgeons and pediatric surgeons.

In addition, based on the score, we propose an algorithm on how to adapt surgery to preoperative cross-sectional imaging. We cannot exclude that subsequent exposures might have influenced visibility and identification of vessels. However, the effect was only marginal when exposure of the major visceral vessels was attempted from both sides of the aorta. As an example, when we left the loops around the coeliac trunk, superior mesenteric artery and left renal artery in place after a previous left-sided exposure, their identification from the right side was only possible after the right-sided medial visceral rotation had been completed.

Today, preoperative analysis of image defined-risk factors at visceral arteries has become a standard. The proposed algorithm for surgical exposure uses this opportunity and goes one step further by translating preoperative imaging into a matching consequential anatomically oriented surgical exposure stratagem. This tactic can be matched to the imaging preoperatively and executed intraoperatively in a standardized way. The semi-quantitative results are one of the key findings of our investigations. For the purpose of practical surgery, however, readers may benefit from the suggested algorithm of tumor resection. In relation to this, we refer to previous publications from Tsuchida [8] and Kiely [6, 50], who recommended and performed comparable exposure techniques, which were highly successful in clinical practice. Our work adds some clear anatomical aspects, which may help to improve planning, performance and safety for retroperitoneal tumor resections. Based on the size of the tumor and its location, auxiliary approaches may be helpful to maintain safety of the major vessels. The thoraco-abdominal approach, based on a lateral thoracotomy, continued into a laparotomy and including incision of the diaphragm, is an excellent exposure technique to obtain control for tumors crossing the thoraco-abdominal junction. An excellent review of the technique in pediatric patients has been provided in the guidelines of the International Society of Paediatric Surgical Oncology (IPSO) Surgical Practice Guidelines [51], in publications of Fuchs et al [52]., Martuciello et al. [53]. , La Quaglia et al. [54]. and Qureshi et al. [55]. Other helpful approaches to improve exposure and control of major visceral vessels like the aorta or the retrohepatic vena cava have been described for trauma surgery [56]. This tactical arsenal described there includes standardized techniques like the mobilization of the liver. The manoeuvers are described in detail in the work of Ciancio et al. [2]. The mobilization of the left liver lobe is especially helpful for tumors in contact with the diaphragm or extending in the interaortocaval area. In cases of encasement of the coeliac trunk, an incision in the right crus of the diaphragm, medial to the esophageal hiatus, allows a safe control of the lower thoracic aorta. An incision of the anterior right-sided diaphragm allows control of the thoracic vena cava, well above the hepatic veins. Finally, the Pringle-manoeuver, consisting of controlling the hepatic artery and the portal vein in the hepato-duodenal ligament might support resection when dealing with a tumor attached to the retrohepatic vena cava.

This approach meets the need to standardize operative techniques, as requested by reference pediatric surgeons [9] and is backed by recent data that surgery plays an important role in improving the outcome of patients with neuroblastoma [13–15, 57].

One limitation of our study was that only adult cadavers were available and that the surgical complexity imposed by tumoral encasement of the vessels could not be directly considered. However, relating the working space to the diameter of the aorta allowed us to assess the view at a specific visceral vessel independent of the size of the abdominal cavity and increased reproducibility. As there is no evidence that relations between aortic diameter and topographic anatomy are different in adult and pediatric patients, the cadaveric setting offers a unique opportunity to gain experience with the concept in adults before operating on pediatric tumors. Cadavers conserved with formaldehyde were more rigid than the fresh frozen cadavers, which imitated more the situation in the clinical operating room. Despite this limitation, we considered the exposure of the vessels to be similar to that of surgery in children. The learning curve of the surgical techniques was not the focus of this study. However, all participating members of the surgical team agreed that the repetition of the standardized steps of the surgical techniques was very beneficial in preparing for major abdominal tumor resections in children.

Surgical technique is recently becoming again a focus of research in neuroblastoma [7, 52, 58, 59] and the present study contributes to experimental evidence on this topic.

Subsequently, we propose that the algorithm be validated in a prospective multicenter trial investigating standardized vs. non-standardized exposure and its impact on complete resection and overall survival of patients with metastatic abdominal neuroblastoma.

## Data Availability

The datasets on which the conclusions of the manuscript rely are all presented in the manuscript.
